# Label‐Free Machine Learning Prediction of Chemotherapy on Tumor Spheroids Using a Microfluidics Droplet Platform

**DOI:** 10.1002/smsc.202500173

**Published:** 2025-07-16

**Authors:** Caroline Parent, Hasti Honari, Tiziana Tocci, Franck Simon, Sakina Zaidi, Audric Jan, Vivian Aubert, Olivier Delattre, Hervé Isambert, Claire Wilhelm, Jean‐Louis Viovy

**Affiliations:** ^1^ CNRS UMR168 Laboratoire Physique des Cellules et Cancer Institut Curie PSL Research University 26 rue d’Ulm 75 005 Paris France; ^2^ INSERM U1330 Children's Oncology Research Unit Research Center PSL Research University Institut Curie 26 rue d’Ulm 75 005 Paris France; ^3^ UAR3750 CNRS Plateforme technologique Institut Pierre Gilles de Gennes PSL Research University 6 rue Jean Calvin 75 005 Paris France

**Keywords:** droplet microfluidics, drug testing, label‐free, machine learning, tumor spheroids

## Abstract

An integrated approach is proposed to rapidly evaluate the effects of anticancer treatments in 3D models, combining a droplet‐based microfluidic platform for spheroid formation and single‐spheroid chemotherapy application, label‐free morphological analysis, and machine learning to assess treatment response. Morphological features of spheroids, such as size and color intensity, are extracted and selected using the multivariate information‐based inductive causation algorithm, and used to train a neural network for spheroid classification into viability classes, derived from metabolic assays performed within the same platform as a benchmark. The model is tested on Ewing sarcoma cell lines and patient‐derived xenograft (PDX) cells, demonstrating robust performance across datasets. It accurately predicts spheroid viability, used to generate dose‐response curves and to determine half maximal inhibitory concentration (IC50) values comparable to traditional biochemical assays. Notably, a model trained on cell line spheroids successfully classifies PDX spheroids, highlighting its adaptability. Compared to convolutional neural network‐based approaches, this method works with smaller training datasets and provides greater interpretability by identifying key morphological features. The droplet platform further reduces cell requirements, while single‐spheroid confinement enhances classification quality. Overall, this label‐free experimental and analytical platform is confirmed as a scalable, efficient, and dynamic tool for drug screening.

## Graphical abstract

A droplet‐based microfluidic platform is used to form and test drugs on spheroids, from cell line and PDX cells. Images are taken and morphological features of spheroids are used to train a neural network to classify spheroid viability in a label‐free manner. Ground truth from metabolic assays validates predictions, enabling accurate drug response profiling from reduced cell quantity.

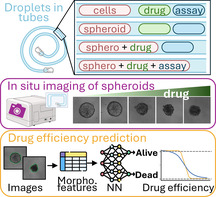

## 1 Introduction

One of the key challenges in oncology is the variability of responses to treatment and the frequent development of resistance to therapy.^[^ ^1^, ^2^, ^3^ ^]^ To tackle these complexities, research is continuously progressing in understanding molecular processes, identifying new therapeutic targets and associated molecular markers, and developing new drugs and therapeutic approaches.^[^ ^4^, ^5^ ^]^ These findings are integrated into the general framework of “precision medicine”.^[^ ^6^ ^]^ Despite such progress, this strategy is not always sufficiently predictive, mainly due to tumor heterogeneity.^[^ ^2^, ^7^ ^]^ A potential alternative or complementary approach would consist of testing drugs in vitro on patient‐derived tumor models to identify the most suitable treatment options for each patient.^[^ ^8^, ^9^ ^]^ This would facilitate the refinement of treatment selection on an individual patient basis (“personalized drug screening”). To be clinically relevant, this drug screening must be standardized, sensitive, and conducted quickly enough to avoid delaying patient treatment.^[^ ^9^, ^10^ ^]^ In addition, it should be achievable with minimal cell quantities to test a wide enough set of drug combinations and concentrations, using the limited number of cells provided by minimally invasive biopsies such as fine needle aspiration.^[^ ^11^ ^]^

The aim of refining treatment selection raises the question of the biological realism of the in vitro model. Conventional cell culture in monolayers at the bottom of culture wells (“2D culture”) has been questioned as real tumors are 3D aggregates with potentially very different metabolic processes and drug permeability.^[^ ^12^, ^13^ ^]^ 3D tumor spheroid models are thus preferred for their ability to better mimic the heterogeneity, morphology, biological mechanisms, and the resistance of native tumors compared to traditional 2D models.^[^ ^14^, ^15^, ^16^, ^17^, ^18^ ^]^

Finally, to allow screening of a reasonable variety of treatment conditions, current 2D or 3D in vitro cell culture methods most often require initial numbers of cells incompatible with most minimally invasive sampling methods. Microfluidic technologies have emerged as valuable tools to overcome this limitation, offering precise control over the cellular microenvironment while minimizing the consumption of cells and reagents in integrated systems.^[^ ^19^, ^20^, ^21^, ^22^, ^23^, ^24^ ^]^ Various microfluidics systems have been developed to perform drug screening on 3D models^[^ ^25^ ^]^ using techniques such as hanging drop culture,^[^ ^26^ ^]^ microwells,^[^ ^27^, ^28^, ^29^, ^30^, ^31^, ^32^, ^33^ ^]^ hydrogel encapsulation,^[^ ^34^, ^35^ ^]^ and droplet‐based systems.^[^ ^36^, ^37^, ^38^, ^39^, ^40^, ^41^, ^42^, ^43^ ^]^

However, using these models in personalized medicine presents challenges, especially when the gold‐standard readout to evaluate cell viability after treatment is to conduct biochemical assays. Cellular assays such as live/dead fluorescent markers, for instance, offer precise results on cell viability, but they require extensive, costly, and time‐consuming image analysis, and are difficult to quantify in 3D.^[^ ^18^, ^44^ ^]^ Additionally, many of these assays, such as the MTT assay (a colorimetric assay for assessing cell metabolic activity) or live/dead, are endpoint assays and may lack precision in cytotoxicity measurement.^[^ ^45^, ^46^ ^]^ Globally, biochemical assays are labor‐intensive in both their implementation and analysis, which hinders their integration into routine treatment protocols.

The present work stems from the observation that spheroids undergo specific morphological changes when exposed to cytotoxic agents, as observed by us and others.^[^ ^47^, ^48^, ^49^, ^50^ ^]^ These changes appear to contain a high amount of information, and this study aims to investigate whether machine learning methods can analyze and interpret these morphological changes, ultimately providing a label‐free, quantitative, robust, and reliable method for assessing the effectiveness of anticancer therapies. Some studies have already explored the use of deep learning in cancer diagnosis and treatment orientation,^[^ ^51^, ^52^, ^53^, ^54^ ^]^ and even for evaluating drug efficacy in tumor spheroids. For example, optical coherence tomography^[^ ^55^, ^56^ ^]^ and refractive index tomography^[^ ^57^ ^]^ have been used to extract features from spheroids exposed to anticancer treatment, but these approaches require sophisticated and specific microscopy instruments and are difficult to scale up for high‐throughput applications. Other approaches have used images of spheroids combined with deep learning algorithms for label‐free analysis. Benning et al.^[^ ^58^ ^]^ and Tröndel et al.^[^ ^59^ ^]^ trained convolutional neural networks (CNNs) to classify spheroids based on their response to drugs. Similarly, Chiang et al.^[^ ^60^ ^]^ developed a CNN model to classify spheroids and predict the drug concentrations to which they had been exposed. Very detailed classification was achieved, for example, as a function of the applied drug concentration, but to our knowledge, no prediction of drug efficacy was obtained. These studies were conducted only on cell lines and focused on classification models trained directly on images.

This article adopts a streamlined and operational approach for the direct label‐free prediction of drug efficacy. It aims for applications in clinical settings or drug development. This approach relies on the morphological analysis of spheroids using a machine learning model. Tumor spheroids were first generated and treated with drugs within droplets, using a previously described microfluidic platform.^[^ ^61^ ^]^ Brightfield images of the spheroids were recorded at several time points during treatment. To enhance image quality and enable automated imaging in controlled exposure conditions, a new “cartridge” was developed for use with a plate imager.

Unlike previous studies, however, this machine learning model was not trained directly on images, which can be sensitive to batch effect, for example, between cell line and patient‐derived specimen, and might not generalize well from small training sets. Instead, we opted for a two‐step approach, starting with a feature selection step before the classification task. To this end, a broad range of morphological features (e.g., size, texture,^[^ ^62^ ^]^ color) were first extracted from the images. Then, the multivariate information‐based inductive causation (MIIC) method^[^ ^63^, ^64^ ^]^ was used to select the most informative features for classifying spheroids, as MIIC tends to outperform deep learning methods in uncovering relevant information from small datasets.^[^ ^65^ ^]^ Finally, the most informative features were used to train a neural network to classify the spheroids into viability classes. As the ground truth for the machine learning model, a fluorescent end point metabolic assay was performed to associate each spheroid with a discrete viability class, used for model training and accuracy assessment. The model was initially trained and tested on spheroids derived from a cell line, and then on spheroids derived from patient‐derived xenografts (PDX). The study also evaluated the ability of a model trained on cell line spheroids to classify PDX spheroids and predict the dose‐response relationship. Using these classifications, “virtual” dose‐response curves were generated, enabling the estimation of half maximal inhibitory concentration (IC50) values based solely on the morphological characteristics of spheroids. Then, to fully exploit the benefits of our platform, spheroid viability was estimated over the drug exposure time. Finally, the same label‐free assay was applied to a dataset issued from another system to investigate the versatility of this approach.

These results demonstrate that the machine learning approach provides comparable insights to traditional biochemical assays while leveraging a label‐free and morphology‐based methodology.

## 2 Results

### 2.1 The Microfluidic Platform Facilitates Spheroid Production and Drug Treatment

Tumor spheroids were generated in droplets confined within tubing, using cells derived either from an established cell line or from a PDX. The PDX samples were derived from Ewing sarcoma, and the chosen cell line for this cancer was A673. Once encapsulated in droplets, the cells in suspension aggregated spontaneously into spheroids in less than 24 h. The droplet‐based system tends to produce uniform spheroids, likely due to continuous agitation during droplet formation and internal recirculation flows, which promote consistent cellular aggregation.

First, the growth and viability of the spheroids in droplets were assessed. For this purpose, PDX‐derived spheroids were measured over a week, and a metabolic assay was performed (see method for details) to measure their metabolic activity over 8 days. Over this period, the spheroids grew continuously. This was confirmed by the increase in their metabolic activity, which doubled each day during the first 3 days. Then, it increased more slowly, and by day 8, the metabolic activity reached 800% of the level observed on day 1 (Figure S1, Supporting Information).

**Figure 1:**
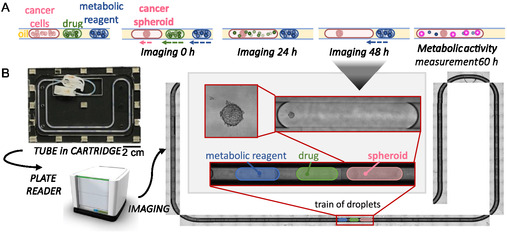
A) Workflow of drug testing in droplets. Trains of three droplets, with cells in suspension, drug solution, and metabolic assay reagent are generated in tubes. After 24 h, the cells have formed a spheroid, and the drug droplet is merged with the spheroid droplet. Spheroids are exposed to the drug for 48 h. Then, as a ground truth for later, the metabolic assay reagent droplet is merged with the spheroid treated with the drug to measure its metabolic activity. The readout is performed after overnight incubation. Images are taken daily. B) System to image the spheroids inside the droplets inside the tubes. The tubes are loaded into a “cartridge” made of two milled COC sheets. The cartridge, sized as a conventional well plate, is then inserted into a plate reader that automatically images the entire tube with the spheroids inside.

After confirming that the spheroids were viable and growing for a period long enough to perform the drug assay (5 days), a drug screening protocol in droplets was established (**Figure** **1**A). The use of 20 replicates per condition minimized variability in initial cell number or morphology and helped isolate drug‐induced effects. Triplicates were performed systematically for both the cell line and the PDX.

Imaging the spheroids inside droplets within tubes presented a significant challenge. To address this, a custom‐made device was designed to fit into a plate reader (Figure 1B). The device consisted of two micromachined cyclic olefin copolymer (COC) sheets: one transparent sheet that allowed imaging through it, while the other featured a channel to securely hold the tube. The two sheets were held together by magnets. To minimize optical aberrations, the same oil used to separate the droplets was introduced between the two sheets. The plate reader was programmed to capture images along the length of the tube at different focal distances.

**Figure 2:**
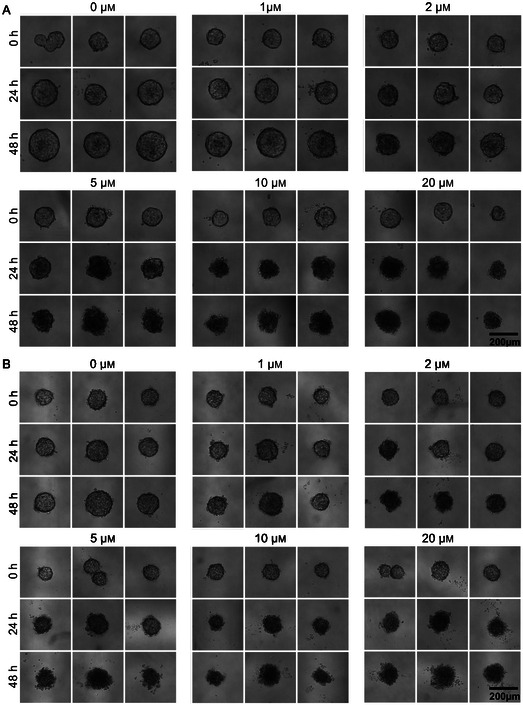
Images of spheroids in droplets derived from A) the cell line and B) the PDX cells, exposed to different drug concentrations (etoposide) after 0, 24, and 48 h of drug exposure. For each drug concentration, the same three spheroids are shown at different time points. Spheroids derived from the cell line are cultivated from ≈700 cells, and the ones from the PDX from 1000 cells, in 1.8 μL droplets.

Images in bright‐field were taken before drug exposure (0 h), after 24 h, and after 48 h of drug exposure. A sample of images is presented for the spheroids derived from the cell line in **Figure** **2**A and the PDX in Figure 2B. In some droplets, several small spheroids were formed before drug addition, but they were fused the next day. It represented 7.5% of the cell line's spheroids and 7.7% of the PDX spheroids. As observable on the images, the spheroids from both cell types undergo morphological changes depending on time and drug concentration. In the no‐drug case, the spheroids increase in size. For increasing drug concentrations, a qualitative change is observed in the morphology between 0 and 48 h, occurring between 2 and 5 μm for the cell line and between 1 and 2 μm for the PDX. The spheroids become darker, smaller, and their texture change. This change of morphology is also visible after 24 h for spheroids submitted to the highest concentrations of drug. The main aim of this article is to determine whether this qualitative observation can be transformed, thanks to machine learning, into a quantitative, reproducible, and predictive tool for drug response prediction.

### 2.2 Morphological Features of Spheroids Evolve During Drug Treatment

After exposing the spheroids to a range of drug concentrations and imaging them at three time points, various morphological features were extracted, including area, circularity, mean gray value, as well as several texture descriptors (homogeneity, energy, and correlation) derived from the computation of the gray level co‐occurrence matrix (GLCM).^[^ ^66^ ^]^ The full list of morphological descriptors is available in Table S1, Supporting Information. Since the microfluidic platform allows the tracking of each spheroid individually, it was possible to obtain the features of individual spheroids over time (Figure S2, Supporting Information). For the (minority) of droplets containing multiple small spheroids, an average of the circularity, mean gray value, and texture descriptors was taken while the area was summed according to the formula given in the methods section.

**Figure 3:**
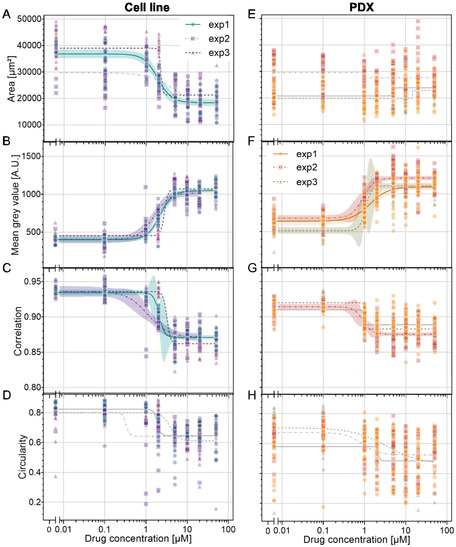
Morphological features of spheroids in droplets derived from A–D) the cell line and E–H) the PDX. Each combination of color and shape represents one independent experiment. Each point represents a single spheroid. The curves represent sigmoid fits with 95% confidence bands. The curves in gray represent curves with poor goodness‐of‐fit (*R* ^2^ < 0.5).

The morphological features—area, circularity, mean gray value, and correlation—after 48 h of drug exposure were then plotted against drug concentration and fitted to a sigmoid dose‐response‐like curve. A selection of these plots is shown in **Figure** **3**A for the cell line and in Figure 3B for the PDX. Additional graphs for the features at 0 and 24 h, as well as other texture descriptors and their variations, are presented in Figure S3 and S4, Supporting Information for the cell line and in Figure S5 and S6, Supporting Information for the PDX. When possible, an IC50 value was derived from the sigmoidal fit, as presented in Figure S7, Supporting Information. For the cell line, several indicators demonstrate concentration‐dependent changes, including spheroid area, mean gray value, and the correlation texture feature. In contrast, for the PDX model, only the mean gray value could be used to fit a sigmoidal dose‐response curve, and even for this feature, the data points are more heterogeneous than for the cell line.

As an intermediate conclusion, morphological analysis alone seems to reveal significant trends upon drug concentration and time of exposure. However, it lacks the precision and reliability needed to accurately assess drug efficacy, especially for PDX, which are the most relevant to clinical settings. A supervised machine learning approach was thus developed to address this limitation. It first involved the establishment of a benchmark as a reference for training.

### 2.3 Dose‐Response Curve, Using a Metabolic Assay, Serves as a Benchmark for Assessing Spheroid Viability

**Figure 4:**
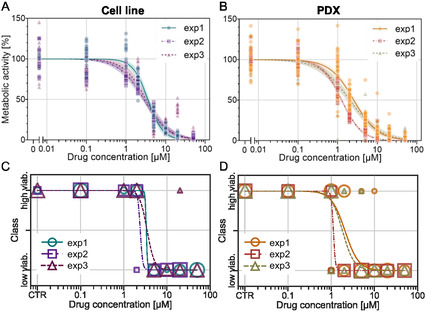
Dose‐response curves with normalized metabolic activity depending on drug concentration (etoposide) of spheroids in droplets derived from A) the cell line and B) the PDX cells. C,D) Dose response curves with discretized metabolic activity into two viability classes depending on drug concentration for the cell line and PDX, respectively. Each combination of color and shape represents one independent experiment. For continuous metabolic activity, each point represents a single spheroid. For discretized metabolic activity, the size of each point on the plot is scaled proportionally to the number of overlapping data points. The curves represent sigmoid fits, with 95% confidence bands for the continuous metabolic activity.

As a benchmark for the machine learning approach, a metabolic bioassay was conducted after 48 h of drug exposure to evaluate the drug's efficacy using a standard method. Each spheroid was then associated with an experimental metabolic activity score, defined as the fluorescence signal normalized to the average of the control (see methods for details). Subsequently, dose‐response curves were generated for each independent experiment, for both the cell line (**Figure** **4**A) and the PDX (Figure 4B). Data were then fitted to a sigmoid curve, and the IC50 values were determined for each experiment. The average IC50 values obtained were IC_50,cellline_ = 3.2 ± 0.3 μm for the cell line and IC_50,PDX_ = 1.9 ± 0.2 μm for the PDX.

This “bioassay‐derived” metabolic activity score exhibits significant heterogeneity for a given drug concentration (e.g., for the cell line, the metabolic activity without drug ranges from 65% to 145%), reflecting the intrinsic uniqueness and variability of living systems. Moreover, this wide variability is also reflected in the morphological features of spheroids, where spheroids with similar metabolic activity display heterogeneous morphological properties (Figure S8, Supporting Information). This variability poses a significant challenge to the algorithm's ability to make meaningful predictions.

To address this issue, we used the experimentally observed fact that drug efficacy presents a relatively sharp threshold, as indicated by the sigmoidal shape of the dose‐response curves. On this basis, spheroids were classified as “low viability” for those with a metabolic activity below 50%, and “high viability” for those with a score above 50%. The dose‐response curves generated after this classification are shown in Figure 4C,D, Supporting Information.

### 2.4 The MIIC Algorithm Helps Identify Relevant Morphological Features

Our objective was to develop a machine learning‐based approach that utilized spheroid morphological properties as input features and predicted drug efficacy as the output.

**Figure 5:**
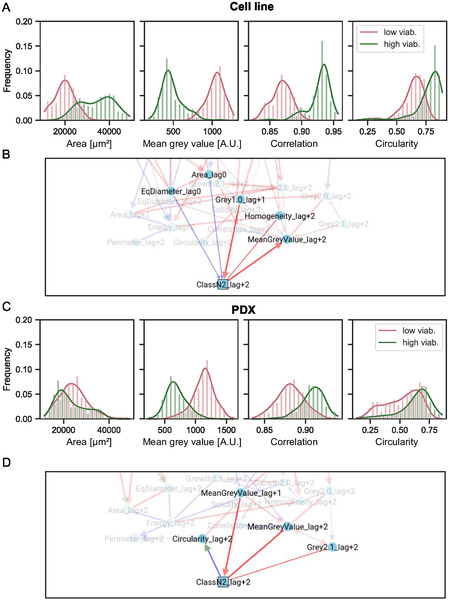
A,C) Histograms of the distribution of four features after 48 h of drug exposure depending on their assigned class for respectively the cell line and the PDX. The curve represents the probability density for each class, bars show the normalized frequency of the data. *N* = 3. B,D) Cropped MIIC network, respectively, for the cell line and the PDX.

To explore the relationship between morphological features and viability classes, the histograms and density distributions of spheroid features of the two cell types according to their assigned classes were plotted in **Figure** **5**A,C. Features for spheroids classified as “high viability” and “low viability” are shown in green and magenta, respectively.

For the cell line, the area, mean gray value, and correlation graphs show minimal overlap between the two classes, suggesting that these features are robust features for classification. However, for the PDX, only the mean gray value graph presents classes with relatively low overlap, while the other plots show substantial overlap between the two classes. This is consistent with the observations in Figure 2, where this gray value seems to provide the most significant evolution with drug concentration. These curves, however, suggest that other features still contain some discriminative power, but provide no clue about the redundancy or independence of the contained information.

To identify the most relevant features for training the machine learning algorithm, the strength of association between each feature and the spheroid viability state was analyzed using the MIIC algorithm, adapted to analyze non‐stationary temporal data. It was applied to the dataset containing all extracted features for each spheroid, measured at different drug exposure times. This analysis computes the mutual information between each feature and the spheroid viability label, quantifying the feature's importance in determining the spheroid class. Additionally, the algorithm retrieved associations between features. Some features are identified as having a direct, possibly causal association with the spheroid class, while other features are identified as having an indirect impact on the spheroid class. The features, their mutual information with the class, and their relationship (direct or indirect) to the class are summarized in **Table** **1**, and the networks highlighting the most informative features derived from the MIIC analysis are shown in Figure 5B,D for the two cell types. The full temporal networks are reported in Figure S9, Supporting Information.

**Table 1:** The mutual information, in bits, shared between the class and the features. The column “direct link” indicates whether there is a link in the graphical network between the class and the feature.

Cell type	Feature	Direct link (Y/N)	Mutual information [bit]
Cell line	Homogeneity 48 h	Y	1.360
	Δ Gray value 24–0 h	Y	0.934
	Mean gray value 48 h	Y	0.885
	Δ Gray value 48–0 h	N	0.862
	Correlation 48 h	N	0.816
	Growth 48–0 h	N	0.786
	Diameter 48 h	N	0.641
	Area 48 h	N	0.641
	Energy 48 h	N	0.636
	Solidity 48 h	N	0.540
	Circularity 48 h	N	0.498
	Perimeter 48 h	N	0.281
	Diameter 0 h	Y	0.028
	Area 0 h	Y	0.028
PDX	Mean gray value 24 h	Y	0.646
	Mean gray value 48 h	Y	0.617
	Δ Gray value 48–0 h	N	0.621
	Δ Gray value 24–0 h	N	0.562
	Correlation 48 h	N	0.376
	Correlation 24 h	N	0.371
	Solidity 48 h	N	0.165
	Circularity 48 h	Y	0.155
	Circularity 24 h	N	0.101
	Perimeter 48 h	N	0.079
	Solidity 24 h	N	0.069
	Δ Gray value 48–24 h	Y	0.065
	Perimeter 24 h	N	0.061
	Area 48 h	N	0.043
	Diameter 48 h	N	0.043

The MIIC network also enabled the identification of more subtle relationships between features. For the cell line, the initial spheroid size (prior to drug exposure) showed a slight influence on the final classification in terms of mutual information. This suggests that smaller spheroids derived from this cell line may exhibit slightly greater sensitivity to the drug compared to larger ones. Additionally, the presence of multiple small spheroids before drug exposure was found to have no measurable impact on spheroid classification, justifying our initial choice of integrating them into the dataset.

The features identified by MIIC as the most critical for spheroid classification are consistent with the qualitative intuitions derived from class overlap. These features display an important variation across classes, but their specific or redundant information about classes is not straightforward to assess. In this context, MIIC analysis offers a quantitative evaluation of each parameter's significance, providing deeper insights into their specific contributions to spheroid classification.

### 2.5 A Supervised Machine Learning Model Classifies Spheroids in Viability Classes Based on their Morphological Features for IC50 Prediction

A supervised machine learning model based on a neural network was then trained to classify the spheroids into two classes, “high viability” and “low viability”. Input features were selected based on their relevance, as identified by the MIIC algorithm. The features selected to train the model were the 15 with the highest weight. For the cell line, as data at 24 h was missing for one of the experiments, only features at 0 and 48 h were selected. Spheroids with missing data at any of the selected time points were excluded from the dataset.

**Figure 6:**
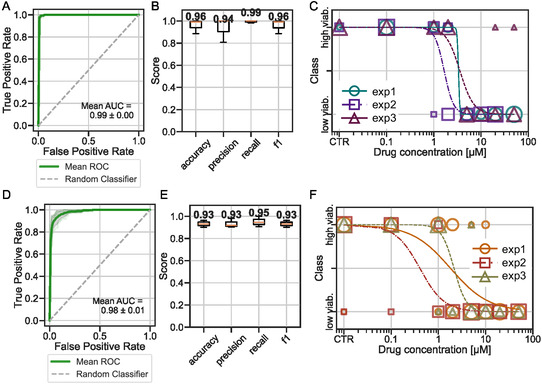
Results of machine learning for A–C) cell line and D–F) PDX. A,D) Averaged ROC curves across cross‐validation folds. B,D) Box plot with average metrics across cross‐validation folds. The box shows the interquartile range (IQR), with the median value indicated as a horizontal line within the box. The whiskers extend to the minimum and maximum values within 1.5 times the IQR. All metrics are computed with respect to the predicted viability class (low vs. high), compared to the ground truth derived from the metabolic activity measured on the same spheroids used for imaging. C,E) Predicted dose‐response‐like curves after classification. Each combination of color and shape represents one independent experiment. The size of each point on the plot is scaled proportionally to the number of overlapping data points.

The algorithm was trained on 2 out of the 3 experiments and tested on the 3rd experiment. The process was repeated for each experiment as a cross‐validation process. The detailed number of points in each class and each experiment is detailed in Figure S10A,B, Supporting Information. To evaluate the performance of the model, the receiver operating characteristics (ROC)–AUC scores are presented in **Figure** **6**A,D, Supporting Information as well as four metrics: accuracy, precision, recall, and f1 score, presented in Figure 6B,E, Supporting Information.^[^ ^67^ ^]^ Essentially, the closer these metrics are to 1, the better the classification performance. For the cell line dataset, the model achieved near‐perfect classification, with an f1 score of 96%. For the PDX, the model performance was slightly lower (f1 score of 93%). The curves representing the accuracy versus epoch are visible in Figure S11, Supporting Information, indicating that the model did not overfit.

**Figure 7:**
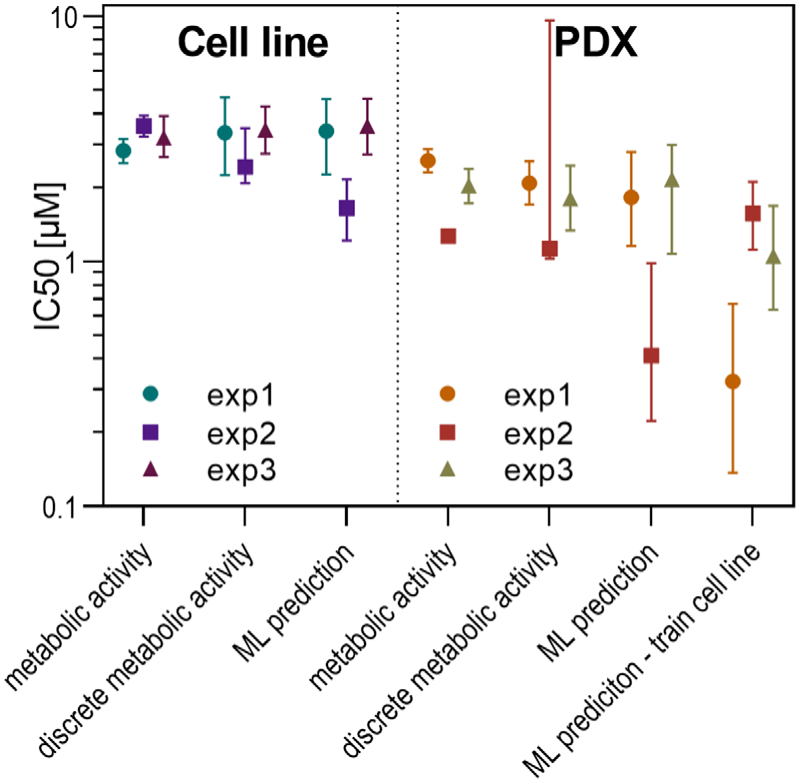
IC50 values computed from metabolic activity and inferred from machine learning classification for the cell line and the PDX. Each point represents the prediction of an independent experiment. The error bars represent the 95% CI.

This classification was then used to estimate drug efficacy. To this end, the predicted class for each spheroid (0 for “low viability” and 1 for “high viability”) was plotted as a function of the drug concentration to which the spheroid was exposed, similar to a dose‐response curve (Figure 6C,F, Supporting Information). The comparison between the IC50 values obtained using the machine learning approach and the metabolic assay approach is shown in **Figure** **7** and **Table** **2**. Indeed, it can be observed that the machine learning method provides an IC50 value close to that of the metabolic assay used as an initial reference.

**Table 2:** IC50 values for the cell line and the PDX computed with different methods.

Cell type	Assay	IC50 [μm]		
		exp1	exp2	exp3
Cell line	Metabolic activity	2.8	3.6	3.2
	Discrete metabolic activity	3.3	2.4	3.4
	Machine learning prediction	3.3	1.7	3.6
PDX	Metabolic activity	2.6	1.3	2.0
	Discrete metabolic activity	2.1	1.1	1.8
	Machine learning prediction	1.8	0.4	2.1
	ML prediction, training cell line	0.3	1.6	1.1

To assess the robustness of our classification into two viability classes, the same procedure was repeated using a “three classes” classification. Spheroids were classified as “low viability” if their score was below 25%, “high viability” if it was above 75%, and “intermediate” for scores in between. The distribution of these classes is shown in Figure S10C,D, Supporting Information. The results regarding viability and IC50 did not differ significantly from those obtained with the two‐class model, despite lower performance metrics, with an f1score of 64% for the cell line and 75% for the PDX model (Figure S12, Supporting Information). This supported our initial decision to use a binary classification model.

One of the key advantages of the droplet platform is its ability to track spheroids over time at a single‐scale level, enabling dynamic assessments of spheroid viability during drug exposure. To explore this potential, the neural network model was trained, as before, to classify spheroids based on their viability class. However, in that case, only the features measured after 48 h were used to train the model. Then, the features at 24 h were used to make predictions on spheroid viability class. From this classification, dose‐response curves and IC50 values were inferred (Figure S13 and Table S3, Supporting Information).

The comparison of IC50 values inferred from morphological features at 24 h with those obtained from the metabolic assay at 48 h revealed no significant differences for the cell line. However, for the PDX, a slight shift of IC50 toward higher concentrations was detected, as expected for spheroids exposed to the drug for a shorter time. These results highlight the platform's potential for dynamic drug sensitivity assessments over time. However, since the metabolic activity scores were only known after 48 h of drug exposure, these predictions could not be validated against a definitive ground truth.

### 2.6 A Model Trained on Cell Line Data Successfully Predicts PDX Response

Finally, to check the robustness and generality of the approach, targeting clinical use, the neural network model was trained using data derived from the cell line spheroids and subsequently tested on the PDX spheroids to evaluate its predictive accuracy in a different biological context. The set of features identified as important for the PDX was used.

**Figure 8:**
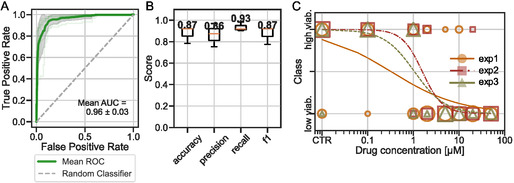
Results of the machine learning algorithm trained on the cell line dataset and tested on the PDX dataset. A) Averaged ROC curve across cross‐validation folds. B) Boxplot with average metrics across cross‐validation folds. The box shows the IQR, with the median value indicated as a horizontal line within the box. The whiskers extend to the minimum and maximum values within 1.5 times the IQR. All metrics are computed with respect to the predicted viability class (low vs. high), compared to the ground truth derived from the metabolic activity measured on the same spheroids used for imaging. C) Predicted dose‐response‐like curves after classification. Each combination of color and shape represents one independent experiment. The size of each point on the plot is scaled proportionally to the number of overlapping data points.

After applying the trained model to the PDX spheroids, the overall accuracy of the predictions was equivalent to the one obtained previously, when training and testing on the PDX dataset. The model's performance is highly satisfactory, with a classification accuracy of 87%, demonstrating a generalization capacity across different sample types (**Figure** **8**).

Moreover, the model demonstrated an ability to predict the drug response profiles of PDX spheroids, identifying patterns of resistance and sensitivity that aligned closely with those observed through biochemical assays. This suggests that despite the inherent biological differences between cell lines and PDX samples, the features learned by the neural network from cell line data could retain relevance in the context of patient‐derived samples.

## 3 Discussion

This study advances the framework of a droplet‐based microfluidic platform for anticancer drug screening in 3D by incorporating a label‐free assay to assess drug efficacy. The long‐term objective is to provide a platform allowing fast, high‐throughput, and label‐free screening of anticancer drugs on tumor spheroids, using a minimal number of cells.

The first challenge encountered was the integration of in situ and brightfield imaging. To address this, a custom tube holder device was designed to be compatible with a commercial automated plate reader, allowing spheroid imaging directly within droplets. This approach has the advantage of using commonly used laboratory equipment. However, it requires an operator to manually launch image acquisition for each tube. Additionally, the plate reader, designed to image evenly spaced wells, captured many unnecessary images without spheroids, requiring preprocessing of images before analysis. A (time‐consuming) manual step was set to select the spheroids among the images, followed by an automated spheroid segmentation. While a future integration of AI for automated spheroid identification could significantly reduce analysis time, we opted for manual selection at this stage to ensure precise identification, particularly for spheroids at the edges of droplets or slightly out of focus. Not all images were usable due to these limitations, but manual sorting ensured reliable tracking of the spheroids. Further development in imaging and tube‐holder design specifically tailored for this droplets in tubes platform will be to fully automate both imaging and analysis, reducing hands‐on time and increasing throughput, to enhance the platform's applicability for pharmaceutical research and clinical applications.

The images obtained from both the cell line and the PDX spheroids treated with the drug suggested, at first glance, a qualitative correlation between their appearance and the amount of drug they have been exposed to. The quantitative analysis revealed that area, mean gray value, and texture correlation varied with drug concentration in the cell line. However, for the PDX model, the data were more heterogeneous, with only the mean gray value showing a significant variation in response to drug concentration. These observations led us to explore a more systematic and robust approach to assess drug efficacy based on morphological features.

For this purpose, we developed a supervised machine learning model to provide quantitative label‐free assessments of drug efficacy. We aimed at minimizing the size of the dataset and the computing power needed for training and operation. For that, unlike prior approaches that directly train the model on images, we chose a two‐step approach, in which various morphological descriptors are first obtained by conventional image analysis and then used in a supervised machine learning approach. Supervised training was based on a metabolic assay that associates a metabolic activity score with each spheroid. To avoid the dispersion associated with the use of a continuous metabolic score variable, and its detrimental effect on the size of the dataset required for training, each spheroid was classified as either 'low viability’ or 'high viability’ based on its metabolic activity score. Each spheroid is then associated with its morphological properties at different drug exposure times and with its viability class. To minimize bias in our relatively small dataset, spheroids were excluded when morphological data were missing at any time point. Nonetheless, data restoration techniques such as interpolation or imputation may offer a valuable strategy for future iterations of the method, particularly in scenarios with sparser sampling.

To better understand how various morphological features contribute to the spheroid's classification, we used a causal network learning algorithm, MIIC, to identify the relationships between the features and their relative importance for classification. After identifying the 15 more relevant features ranked by the MIIC, we trained a neural network model using these features to predict spheroid viability class. The model was first trained and tested on a cell line dataset and then on a PDX dataset, both from Ewing sarcoma.

Our model successfully classified spheroids into two classes with an accuracy of 96% and 93% with a dataset of 316 and 428 data points for the cell line and the PDX, respectively. Other machine learning models were also tested, showing similar performance (Figure S14, Supporting Information). This accuracy is higher than the one reported in Benning et al.^[^ ^58^ ^]^ (90%) and close to the one reported by Tröndle et al.^[^ ^59^ ^]^ (98.2%). However, the performance was achieved here with a smaller dataset, in contrast to direct deep learning from images, which are typically trained on larger image datasets (e.g., for instance *n* = 4974 for Tröndle et al., *n* = 1200 for Benning et al.). The ability to use a relatively small dataset makes this approach more suitable for clinical applications for precision medicine, where the number of available cells from a biopsy, and thus the number of possible tests, is often limited.

Importantly, the label‐free assay developed in this work has the potential to be adapted for broader applications beyond this platform and integrated into other systems. To evaluate the approach's independence from the experimental method, the algorithm was trained and tested on spheroids formed in agarose microwells (see Figure S15 and S16, Supporting Information). The classification f1 score reached 89% for the cell line and 81% for the PDX (Figure S17, Supporting Information). Although this score is lower than that achieved in droplets, it remains highly acceptable. This demonstrates that the strategy, based on feature extraction and neural network classification, can be applied to other experimental platforms, provided they produce high‐quality images of individual spheroids. However, the final accuracy is platform‐dependent. Systems such as the droplet platform, with high spheroid‐to‐spheroid reproducibility in terms of growth, drug exposure, and screening conditions, are expected to yield better results, as shown in this proof of concept.

Remarkably, the reliable classification achieved when training on the cell line dataset and testing on the PDX dataset opens up the possibility of ultimately predicting drug efficacy in primary patient‐derived cells using a label‐free approach. To further validate this objective, the next steps involve expanding the approach by training models of a broader range of PDX spheroids across different cancer types and assessing that they share sufficiently similar morphological properties.

Finally, the predicted class of each spheroid was plotted against drug concentration, and a sigmoid curve was fitted to compute a “morphological” IC50 value. The comparison of IC50 values derived from the metabolic assay and the machine learning model yielded consistent results. Misclassified spheroids were typically those exposed to drug concentrations near the IC50, where an intermediate state might exist, with dying spheroids not yet exhibiting the same features as fully dead ones. We explored this hypothesis by adding an intermediate state between ‘alive’ and ‘dead’, but the results showed poor classification accuracy. This may be explained by the steep decrease in metabolic activity around the IC50 in our dataset. In cancers with a less sharp drug response, a three‐class classification might better capture the spheroid states.

Several other label‐free and real‐time methods have been developed in other teams to assess cell viability.^[^ ^68^, ^69^, ^70^ ^]^ For example, electrical impedance measurement has been successfully applied to monitor spheroid viability by detecting changes in electrical properties, offering a noninvasive and dynamic readout.^[^ ^71^ ^]^ Optical techniques also hold promise: dynamic optical coherence tomography (D‐OCT) enables high‐resolution, time‐dependent monitoring of viability in spheroids by capturing fluctuations in refractive index.^[^ ^72^, ^73^ ^]^ Similarly, Raman spectroscopy and phase contrast imaging have been explored for viability assessment at the single‐cell level, with potential extension to 3D aggregates.^[^ ^74^ ^]^ Although some of these methods involve more complex instrumentation or setup, particularly in the context of 3D systems, they represent valuable complementary strategies. In the future, combining such techniques with our morphological analysis pipeline may offer a more comprehensive assessment of spheroid viability and treatment response.

It should be noted that in vitro assays serve only as indicators of drug efficacy, so their transposition to clinical settings requires an adaptation for each specific situation.

The droplet platform offers high adaptability, making it a promising tool for precision medicine. First, future adaptations could include periodic merging of fresh medium droplets or increasing initial droplet volumes, extending the platform's use to long‐term experiments while maintaining its label‐free nature. In addition, moving toward clinical applications will require an increasing throughput. In this regard, the droplet platform is well‐suited for high‐throughput operation through parallelization across multiple tubes, enabling simultaneous testing without sacrificing the reliability of the droplet merging.

While the combination of this label‐free assay and our droplet platform demonstrates strong synergy, we believe this method holds the potential for broader application to datasets generated using other systems. For example, we successfully applied the same label‐free classification approach to images of spheroids grown, treated, and imaged in an agarose microwell system, achieving reliable accuracy. This highlights the versatility of our method for use in various experimental settings.

Although the current model was trained on Ewing sarcoma spheroids, the approach was designed with generalizability in mind. By focusing on morphological features and their evolution over time rather than on raw image data, we aimed to capture patterns that may be shared across multiple tumor types.

## 4 Conclusion

This study demonstrates the potential of machine learning in developing a label‐free assay for evaluating drug efficacy in cancer spheroids.

First, spheroid morphology was shown to correlate with viability after chemotherapy treatment. However, direct inference of drug efficacy from these observations lacks robustness and consistency across datasets. To address this, we developed a machine learning approach, able to better capture information embedded in spheroid appearance. We extracted morphological features such as size and color from bright‐field images, selected the most relevant ones using the MIIC model, and used these key features to train a neural network to classify the spheroids based on their viability before generating drug‐response curves and IC50 estimations.

This approach offers a simple and robust method for assessing drug treatment efficacy. It outperforms or matches approaches based on deep learning models directly trained on spheroid images, while using less than 500 spheroids. Additionally, it allows for a deeper understanding of the morphological features driving spheroid classification. This insight paves the way for its broad applicability across different cancer types or drug screening systems, using the same machine learning model for diverse datasets.

The integration of this label‐free approach with a droplet‐based microfluidic platform offers a powerful tool for drug screening. The droplet platform ensures reproducible spheroid generation and testing conditions while minimizing cells, a key advantage for rare cancers, minimally invasive biopsies, large‐scale drug screening, and routine clinical workflow. Furthermore, its compatibility with plate readers ensures consistent imaging conditions.

In summary, this approach combines simplicity, efficacy, and broad applicability, offering significant potential for drug screening in cancer research and clinical applications.

## 5 Experimental Section

5.1

5.1.1

##### Cell Line Culture

The Ewing sarcoma A673 cell line (ATCC CRL‐1598) was cultured at 37 °C and 5% CO_2_ in Dulbecco's modified Eagle's medium (DMEM, Gibco, 61965‐026), supplemented with 10% fetal bovine serum (FBS, Dutscher, S1900‐500C) and 1% penicillin/streptomycin (Gibco, 15140‐122). Mycoplasma testing was conducted every from 1 to 2 months.

##### PDX Cells

PDX of Ewing sarcoma (IC‐pPDX‐87) were provided by INSERM U1330 (authorization APAFIS #43745‐2023060615213570 v2, 24/06/2023 given by National Authority). The tumors were dissected and dissociated to obtain a cell suspension of cancer cells. The dissociation was performed as previously described in Buchou et al.,^[^ ^75^ ^]^ in RPMI (Sigma, R8758) using 150 μg mL^−1^ Liberase (Roche, 5 401 020 001) and 150 μg mL^−1^ DNase (Sigma, DN25‐100MG), for 30 min at 37 °C with gentle mixing. Cellular viability was quantified using a Vi‐Cell XR Viability Analyzer (Beckman Coulter, Brea, CA, USA). Once collected, the cells were diluted at the desired concentration in DMEM‐F12 (Gibco, 31 331‐028) supplemented with 2% B27 (Gibco, 17 504 044) and 1% Penicillin/Streptomycin, and used in the few hours following the dissection.

##### Droplet Microfluidics Platform

The droplet microfluidic platform used for generating and culturing tumor spheroids, as well as for conducting drug screening, is detailed in Parent et al.^[^ ^61^ ^]^ Briefly, droplets were formed within PTFE tubing (Adteck Polymer Engineering, BIOBLOCK/14) of internal diameter 0.81 mm and 50 cm long. The droplets were automatically aspirated using a syringe pump (Tecan Systems, Cavro XMP 6000) that pipettes the different solutions from a well plate into the tubes. The system was capable of simultaneously producing eight tubes, each containing 20 trains of three droplets, with each tube corresponding to a different drug concentration.

Each droplet train consisted of one cell suspension droplet (1.8 μL), one drug solution droplet (1.2 μL), and one metabolic activity assay droplet (1.2 μL), separated by oil phase containing surfactant (Fluigent, dSurf, 2% surfactant in HFE oil) with volumes of 0.45, 0.9, respectively, and 1.0 μL between two trains of droplet. The working oil was FC‐40 oil (Merk, F9755), present at the tube extremities. The biocompatible oil (dSurf) was separated from the FC‐40 oil with a droplet of PBS (Sigma‐Aldrich, D8537) of 6 μL.

After droplet formation, the tubes were clamped at both ends and incubated in a black box to protect them from light.

The droplets can be merged on demand to implement protocols with several steps of reagent addition. The merging principle is detailed in Parent et al.^[^ ^61^ ^]^ and Ferraro et al.^[^ ^76^ ^]^ Briefly, at a sufficiently high flow rate, smaller droplets move faster than larger ones, enabling the adjacent droplets to get closer together and then to merge them. In practice, the tubes were plugged back onto the syringe pumps. Then, the droplets were moved at 6 μL s^−1^ for 45 μL, then brought back to the initial position at 0.3 μL s^−1^. This process was repeated to achieve droplet‐to‐droplet contact, and merging was induced by an electric field. As previously discussed,^[^ ^61^ ^]^ some droplet pairs occasionally failed to merge. These unmerged droplets were easily identified in the imaging process and discarded from the analysis.

##### Spheroid Production, Drug Treatment, and Drug Efficacy Readout in Droplet

The droplets in tubes were generated as described before. For experiments with cell line A673, cells were diluted at 400 000 cells mL^−1^, and for experiments with the PDX, at 550 000 cells mL^−1^ to reach ≈700 cells and 1000 cells per droplet, respectively. The drug, etoposide (GreenPharma, Prestw‐396), was diluted in the same culture media as the cells (but not supplemented for the PDX experiment) at seven different concentrations between 0.1 and 50 μm. The metabolic assay, Alamar Blue (Invitrogen, A50101), was diluted in cell culture media to reach a final concentration of 20% (%v/v) after droplet merging.

After 24 h of culture, spheroids were treated with the drug by merging the droplet containing the spheroids with an adjacent droplet containing the drug. After 48 h of treatment, the droplets containing the metabolic assay were merged with those containing the drug‐treated spheroids.

For both the cell line and the PDX, three independent experiments were performed at different times. For the cell line experiment, one experiment consisted of testing eight conditions (no drug and seven drug concentrations) with a maximum of 20 replicates per condition, depending on the droplet merging. For the PDX experiment, one experiment consisted of testing eight conditions two times, so a maximum of 40 replicates per condition.

##### Metabolic Activity Measurement for Spheroid Viability Assessment

After one night of exposure to the metabolic assay, a readout was performed. AlamarBlue is based on resazurin, which is reduced into a fluorescent species in contact with viable cells, leading to an increase in the droplet fluorescence depending on the metabolic activity of the spheroid inside this droplet. The fluorescence of all the tubes was measured with a scanner (Typhoon FLA 9000, filter Cy3, photomultiplier value of 250 V, pixel size of 25 μm).

For each independent experiment, the average fluorescent intensity of each droplet was calculated from the fluorescent image using a Python script (v3.12.7). It was then normalized by subtracting the basal response and dividing by the mean fluorescence of the control droplets (without drug treatment) to obtain a normalized metabolic activity value, as follows in Equation (1)

(1) $$\%\text{metabolic activity}=\frac{I-B}{\frac{1}{N}\sum_{i=1}^{N}\left(I_{0,i}-B\right)}\times 100$$

where *B* corresponded to the basal signal of Alamar alone (without cells), *I*_*0,i*_ to the fluorescence intensity of a control droplet (no drug case) and *I* to the fluorescence intensity of the droplet.

The dose‐response curve for each experiment was plotted with GraphPadPrism (version 9.3.1) and a sigmoid fit was performed using an [inhibitor] versus normalized response with a variable slope, corresponding to the Equation (2)

(2) $$Y=\frac{100}{1+{\frac{{\text{IC}}_{50}}{X}}^{\text{HillSlope}}}$$

where IC_50_ and Hill slope are features estimated from the fit, and *X* are the values of metabolic activity.

##### Image Acquisition

Images of the spheroids were taken with a plate reader (Perkin Elmer, EnSight), equipped with a 4X objective. Each tube was installed into a home‐made device consisting of two COC sheets. The two sheets were maintained together with 17 pairs of magnets (Supermagnete, Q‐06‐04‐02‐HN and Q‐07‐06‐1.2‐N) distributed along the sheets. The tube was inserted into the designed sheet (Figure S18, Supporting Information), and HFE oil (Fluorochem, F051243) was inserted between the two sheets. The plate reader was programmed to take images along the tube at different focal distances. Images were taken every 25 μm, in brightfield, and the parameters of illumination were fixed to 6% of power and 6 ms of exposure time.

##### Image Analysis and Spheroid Segmentation

As the imaging system took pictures of the entire tube, many of the images did not contain spheroids. To address this, a custom program was developed to allow users to quickly identify the locations of the spheroids. The program then cropped the image stack around the spheroid and selected the best image in the focal plane by choosing the image with the highest sharpness, given by the variance of the Laplacian. Segmentation was performed using the Canny Edge algorithm. A binary mask for each spheroid was extracted from which various features were computed: area, circularity, as well as colorimetric and texture features.

The area was calculated as the number of pixels contained within the mask and converted to μm^2^ using the image resolution (1 pixel = 3.26 μm). Circularity was determined using the Equation (3)

(3) $$\text{Circularity}=\frac{4\times \pi \times \text{area}}{{\text{perimeter}}^{2}}$$

The mean gray value was measured within the spheroid, excluding the edges of the spheroid, and the background value was subtracted. The background value was computed by averaging pixel values outside the spheroid mask.

Texture features were extracted using GLCM analysis,^[^ ^62^, ^66^ ^]^ focusing on three key parameters: homogeneity, energy, and correlation. GLCM is a statistical tool that quantifies the spatial relationships between pixel intensities, providing insights into the underlying structural patterns of the texture. Homogeneity indicates the closeness of GLCM elements to the diagonal, reflecting texture uniformity—higher values represent smoother textures. Energy (also known as angular second moment) evaluates the sum of squared GLCM elements, representing texture uniformity and increasing in images with repetitive or regular patterns. Correlation measures the linear dependance between pixel values at specified offsets, capturing the complexity of texture through pixel similarity with neighboring pixels.

The evolution of some parameters over time was also studied by computing the difference in values before and after drug exposure.

Some droplets before drug addition contained several small spheroids that fused latter. In these cases, the spheroid properties were either averaged (for textural, colorimetric parameters, and circularity) or combined for area and diameter using Equation (4)

(4) $${\text{Area}}_{\text{summed}}=\sum_{i=0}^{n}{\left({\text{Area}}_{i}^{3/2}\right)}^{2/3}$$

##### MIIC

The MIIC algorithm is a network reconstruction method to analyze large‐scale biological or biomedical data,^[^ ^64^ ^]^ which has recently been extended to analyze morphodynamic features extracted from time‐lapse images of cellular systems.^[^ ^63^ ^]^ In the present study, this temporal algorithm (tMIIC) was adapted to analyze nonstationary temporal datasets and used as a feature selection approach to identify the most informative morphological features to predict the viability of the spheroids after 48 h in the presence of the drug. MIIC aims to learn a large class of causal and noncausal graphical models, including the effects of unobserved latent variables, corresponding to the presence of unobserved common causes and represented by a bidirected dashed edge. The nonstationary temporal version of the algorithm tMIIC was used to reconstruct the temporal network starting from the spheroids’ temporal data. The algorithm can handle missing data with no need for imputation. The number of temporal layers was set to 3, as each temporal layer corresponds to a different day of image acquisition and feature extraction. The algorithm outputs a network (Figure S9, Supporting Information), where each node represents a variable and each edge encodes a dependency possibly lagged between the 3 time points (0, 24, and 48 h). An edge with an arrow stands for causal relations, while an edge without an arrow stands for association (i.e., its causal or noncausal nature cannot be determined from the available data). Green arrows stand for genuine cause‐effect relations, while an arrow that is not green stands for a putative causal effect, which can either be a genuine causal relation or the effect of a latent variable (i.e., a bidirected edge), although this cannot be determined from the available data.^[^ ^64^ ^]^ The colors of the edges are based on Spearman's correlation coefficient computed for the two linked variables. The color red stands for positive correlation, and the color blue for negative correlation. The variables listed in Table 1 are those sharing the highest mutual information measured in bits. The letter “Y” in the “direct link” column corresponds to a direct effect, as the class and the features are linked in the graph. The letter “N” corresponds to an indirect effect, as the class and the features are not directly linked, but they are significantly associated in terms of shared mutual information. A Python script was used to prepare the data in the format needed by the MIIC algorithm. The MIIC method used is implemented in R.

##### Machine Learning Model

A neural network model was created to assess a supervised classification task. The input was the morphological features of the spheroids at different time points. The target was the class of the spheroid, encoded from its metabolic activity score.

The neural network model was implemented in Python (v3.12.7) using the Keras library. It consisted of three dense layers, starting with an input layer that accepted vectors of dimensionality matching the size of the input data. The first hidden layer comprised 128 units and employed the rectified linear unit (ReLU) activation function to introduce nonlinearity, allowing the model to capture complex patterns in the data. This was followed by a second hidden layer with 10 units, also using the ReLU activation function, designed to further reduce the dimensionality of the input while preserving important features for classification. The output layer contained a number of neurons corresponding to the total number of target classes, and a softmax activation function was applied to produce a probability distribution over these classes. The model was compiled using the Adam optimizer. The loss function was set to categorical cross‐entropy, which is well‐suited for classification tasks involving mutually exclusive classes. The model's performance was tracked using accuracy as the primary metric, enabling the assessment of its classification performance during training and validation.

As each dataset (cell line and PDX) was composed of three independent experiments, a cross‐validation approach was used where the model was trained on two independent experiments and tested on the third experiment, and so on for each of the three experiments.

Spheroids with missing data for one or more features were excluded from the dataset. For the A673 cell line, the dataset was composed of 316 spheroids, and for the PDX, of 428 spheroids. The detailed dataset descriptions are provided in the supplementary material (Figure S10, Supporting Information).

To assess the performance of the model, different metrics were measured for each training/testing set. First, the ROC curve was computed and its area under the curve (ROC–AUC) measured. Then, the accuracy, the precision, the recall, and the f1 score were computed to get insight into the model's performance. Finally, during training, accuracy and loss were computed at each epoch for both the training and test datasets. These metrics were monitored to assess model performance and detect overfitting.

##### Inference of the IC50 from the Machine Learning Class Prediction

For each independent experiment, after the classification of the spheroids with the machine learning model, the predicted class of each spheroid, depending on the drug concentration it had been exposed, was plotted. A sigmoid curve with a variable slope, constrained to plateau between 0 and 1, was fitted to these points, and an IC50 value was inferred for each experiment.

A Bayesian inference approach was used to estimate the IC50 and slope parameters of the dose‐response curve, using the Markov Chain Monte Carlo (MCMC) sampler from the Python package emcee.^[^ ^77^ ^]^ A sigmoid model was fitted to the points. IC50 and slope were assigned uniform priors within the ranges 0–20 and −50 to −0.1, respectively. The likelihood function was defined based on a logistic model for binary classification. The MCMC sampler was initialized with 32 walkers and run for 500 iterations following a burn‐in phase, generating posterior distributions for the parameters. Median parameter values and 95% credible intervals were calculated from the posterior samples.

##### Statistical Analysis

For all experimental dose‐response experiments (metabolic activity and morphological features), data points represent individual spheroids from three independent experiments, with each experiment using distinct color and symbol coding in the plots. Per drug condition, the number of data points ranged from 4 to 19 for the cell line and from 3 to 31 for the PDX models. A sigmoidal dose‐response curve was fitted using GraphPad Prism (version 9.3.1), and 95% CI were displayed as shaded areas around the fitted curves. The metabolic data were normalized following Equation (1). IC50 values were derived from the fitted curves and reported along with their corresponding 95% CI.

For predicted dose‐response curves issued from spheroid classification, the same color and symbol coding were used for each experiment. A sigmoidal dose‐response curve was fitted as explained before, and parameter estimates with 95% CI as IC50 were extracted from the posterior distribution of the fitted model.

All data visualization and curve fitting were conducted using GraphPad Prism or custom scripts in Python (version 3.12.7).

## Conflict of Interest

The authors declare no conflict of interest.

## Author Contributions

**Caroline Parent** and **Hasti Honari**: performed the experiments, analyzed the data and implemented the algorithms. **Caroline Parent**: developed experimental protocols and methodology. **Tiziana Tocci**, **Franck Simon** and **Hervé Isambert**: performed the MIIC analysis. **Audric Jan**: created the device for imaging. **Sakina Zaidi** provided PDX cells. **Olivier Delattre**, **Claire Wilhelm** and **Jean‐Louis Viovy**: conceptualized the project. **Caroline Parent**, **Hasti Honari** and **Jean‐Louis Viovy**: wrote the initial draft. All the authors reviewed and approved the manuscript. **Caroline Parent** and **Hasti Honari** contributed equally as first authors and **Claire Wilhelm** and **Jean‐Louis Viovy** contributed equally as last authors.

## Supporting information

Supplementary Material

## Data Availability

The codes and raw data used in this study are openly available in GitHub repository at https://github.com/cparent3/spheroid_morpho_ML and https://github.com/miicTeam/miic_R_package/tree/tmiic. The data that support the findings of this study are available from the corresponding authors upon reasonable request.

## References

[1] S. Ramón , Y. Cajal , M. Sesé , C. Capdevila , T. Aasen , L. De Mattos‐Arruda , S. Diaz‐Cano , J. Hernández‐Losa , J. Castellví , J. Mol. Med. 2020, 98, 161. 31970428 10.1007/s00109-020-01874-2 PMC7007907

[2] I. Dagogo‐Jack , A. T. Shaw , Nat. Rev. Clin. Oncol. 2018, 15, 81. 29115304 10.1038/nrclinonc.2017.166

[3] M. Jamal‐Hanjani , S. A. Quezada , J. Larkin , C. Swanton , Clin. Cancer Res. 2015, 21, 1258. 25770293 10.1158/1078-0432.CCR-14-1429 PMC4374162

[4] R. Santos , O. Ursu , A. Gaulton , A. P. Bento , R. S. Donadi , C. G. Bologa , A. Karlsson , B. Al‐L azikani, A. Hersey, T. I. Oprea, J. P. Overington Nat. Rev. Drug Discov. 2017, 16, 19. 27910877 10.1038/nrd.2016.230 PMC6314433

[5] X.‐P. Duan , B.‐D. Qin , X.‐D. Jiao , K. Liu , Z. Wang , Y.‐S. Zang , Sig. Transduct. Target Ther. 2024, 9, 57. 10.1038/s41392-024-01760-0 PMC10912713 38438349

[6] N. B. La Thangue , D. J. Kerr , Nat. Rev. Clin. Oncol. 2011, 8, 587. 21862978 10.1038/nrclinonc.2011.121

[7] I. F. Tannock , J. A. Hickman , N Engl. J. Med. 2016, 375, 1289. 27682039 10.1056/NEJMsb1607705

[8] V. Gambardella , N. Tarazona , J. M. Cejalvo , P. Lombardi , M. Huerta , S. Roselló , T. Fleitas , D. Roda , A. Cervantes , N. Tarazona , J. M. Cejalvo , P. Lombardi , M. Huerta , S. Roselló , T. Fleitas , D. Roda , A. Cervantes Cancers 2020, 12, 1009. 10.3390/cancers12041009 PMC7226371 32325878

[9] A. W. J. van Renterghem , J. van de Haar , E. E. Voest , Nat. Rev. Clin. Oncol. 2023, 20, 305. 36914745 10.1038/s41571-023-00745-2

[10] C. Hatzis , P. L. Bedard , N. J. Birkbak , A. H. Beck , H. J. W. L. Aerts , D. F. Stern , L. Shi , R. Clarke , J. Quackenbush , B. Haibe‐Kains , Cancer Res. 2014, 74, 4016. 25015668 10.1158/0008-5472.CAN-14-0725 PMC4119520

[11] B. Franzén , G. Auer , R. Lewensohn , Mol. Oncol. 2024, 18, 2612. 38519839 10.1002/1878-0261.13640 PMC11547246

[12] F. Pampaloni , E. G. Reynaud , E. H. K. Stelzer , Nat. Rev. Mol. Cell Biol. 2007, 8, 839. 17684528 10.1038/nrm2236

[13] J. C. Fontoura , C. Viezzer , F. G. dos Santos , R. A. Ligabue , R. Weinlich , R. D. Puga , D. Antonow , P. Severino , C. Bonorino , Mater. Sci. Eng. C 2020, 107, 110264. 10.1016/j.msec.2019.110264 31761183

[14] A. S. Nunes , A. S. Barros , E. C. Costa , A. F. Moreira , I. J. Correia , Biotechnol. Bioeng. 2019, 116, 206. 30367820 10.1002/bit.26845

[15] S. El Harane , B. Zidi , N. El Harane , K.‐H. Krause , T. Matthes , O. Preynat‐Seauve , Cells 2023, 12, 1001. 37048073 10.3390/cells12071001 PMC10093533

[16] J. He , C. Zhang , A. Ozkan , T. Feng , P. Duan , S. Wang , X. Yang , J. Xie , X. Liu , Mechanobiol. Med. 2023, 1, 100014. 40395637 10.1016/j.mbm.2023.100014 PMC12082161

[17] D. G. Nair , R. Weiskirchen , Curr. Issues Mol. Biol. 2025, 47, 7. 10.3390/cimb47080578 PMC12384976 40864732

[18] S. J. Han , S. Kwon , K. S. Kim , Cancer Cell Int. 2021, 21, 152. 33663530 10.1186/s12935-021-01853-8 PMC7934264

[19] S. Sart , G. Ronteix , S. Jain , G. Amselem , C. N. Baroud , Chem. Rev. 2022, 122, 7061. 35179881 10.1021/acs.chemrev.1c00666

[20] F. Eduati , R. Utharala , D. Madhavan , U. P. Neumann , T. Longerich , T. Cramer , J. Saez‐Rodriguez , C. A. Merten , Nat. Commun. 2018, 9, 2434. 29934552 10.1038/s41467-018-04919-w PMC6015045

[21] A. Tevlek , S. Kecili , O. S. Ozcelik , H. Kulah , H. C. Tekin , ACS Omega 2023, 8, 3630. 36743071 10.1021/acsomega.2c06052 PMC9893254

[22] T. Moragues , D. Arguijo , T. Beneyton , C. Modavi , K. Simutis , A. R. Abate , J.‐C. Baret , A. J. deMello , D. Densmore , A. D. Griffiths , Nat. Rev. Methods Primers 2023, 3, 1.

[23] J. Lin , Y. Hou , Q. Zhang , J.‐M. Lin , Lab Chip 2025, 25, 787. 39774470 10.1039/d4lc00646a

[24] Y. Hou , Y. Zheng , X. Zheng , Y. Sun , X. Yi , Z. Wu , J.‐M. Lin , Lab Chip 2023, 23, 2654. 37190976 10.1039/d3lc00251a

[25] B. D. Cardoso , E. M. S. Castanheira , S. Lanceros‐Méndez , V. F. Cardoso , Adv. Healthcare Mater. 2023, 12, 2202936. 10.1002/adhm.202202936 PMC11468737 36898671

[26] A. A. Popova , T. Tronser , K. Demir , P. Haitz , K. Kuodyte , V. Starkuviene , P. Wajda , P. A. Levkin , Small 2019, 15, 1901299. 10.1002/smll.201901299 31058427

[27] B. Patra , C.‐C. Peng , W.‐H. Liao , C.‐H. Lee , Y.‐C. Tung , Sci. Rep. 2016, 6. 10.1038/srep21061 PMC4753452 26877244

[28] B. Schuster , M. Junkin , S. S. Kashaf , I. Romero‐Calvo , K. Kirby , J. Matthews , C. R. Weber , A. Rzhetsky , K. P. White , S. Tay , Nat. Commun. 2020, 11. 10.1038/s41467-020-19058-4 PMC7573629 33077832

[29] P. Bregigeon , C. Rivière , L. Franqueville , C. Vollaire , J. Marchalot , M. Frénéa‐Robin , Lab Chip 2022, 22, 2489. 35475509 10.1039/d2lc00074a

[30] T. D. Do , U. T. Pham , L. P. Nguyen , T. M. Nguyen , C. N. Bui , S. Oliver , P. Pham , T. Q. Tran , B. T. Hoang , M. T. H. Pham , D. T. N. Pham , D. T. Nguyen , Diagnostics 2023, 13. 10.3390/diagnostics13081394 PMC10137404 37189495

[31] N. Sandström , V. Carannante , K. Olofsson , P. A. Sandoz , E. L. Moussaud‐Lamodière , B. Seashore‐Ludlow , H. Van Ooijen , Q. Verron , T. Frisk , M. Takai , M. Wiklund , P. Östling , B. Önfelt , Cell Rep. Methods 2022, 2, 100256. 35880015 10.1016/j.crmeth.2022.100256 PMC9308168

[32] D. Dorrigiv , P.‐A. Goyette , A. St‐Georges‐Robillard , A.‐M. Mes‐Masson , T. Gervais , Cancers 2023, 15, 1060. 36831403 10.3390/cancers15041060 PMC9954565

[33] M. Liu , Y. Wang , C. Wang , P. Li , J. Qiu , N. Yang , M. Sun , L. Han , Adv. Healthcare Mater. 2024, 13, 2402321. 10.1002/adhm.202402321 39126126

[34] L. Yu , M. C. W. Chen , K. C. Cheung , Lab Chip 2010, 10, 2424. 20694216 10.1039/c004590j

[35] E. Prince , S. Kheiri , Y. Wang , F. Xu , J. Cruickshank , V. Topolskaia , H. Tao , E. W. K. Young , A. P. McGuigan , D. W. Cescon , E. Kumacheva , Adv. Healthcare Mater. 2022, 11, 2101085. 10.1002/adhm.202101085 34636180

[36] P. Sabhachandani , V. Motwani , N. Cohen , S. Sarkar , V. Torchilin , T. Konry , Lab Chip 2016, 16, 497. 26686985 10.1039/c5lc01139f PMC4834071

[37] M. Courtney , X. Chen , S. Chan , T. Mohamed , P. P. N. Rao , C. L. Ren , Anal. Chem. 2017, 89, 910. 27959505 10.1021/acs.analchem.6b04039

[38] R. F.‐X. Tomasi , S. Sart , T. Champetier , C. N. Baroud , Cell Rep. 2020, 31, 107670. 32460010 10.1016/j.celrep.2020.107670 PMC7262598

[39] R. Fevre , G. Mary , N. Vertti‐Quintero , A. Durand , R. F.‐X. Tomasi , E. Del Nery , C. N. Baroud , iScience 2023, 26, 106651. 37168549 10.1016/j.isci.2023.106651 PMC10165258

[40] E. Steinberg , R. Friedman , Y. Goldstein , N. Friedman , O. Beharier , J. A. Demma , G. Zamir , A. Hubert , O. Benny , Commun. Biol. 2023, 6. 10.1038/s42003-023-05531-5 PMC10643569 37957280

[41] J. Clausell‐Tormos , D. Lieber , J.‐C. Baret , A. El‐Harrak , O. J. Miller , L. Frenz , J. Blouwolff , K. J. Humphry , S. Köster , H. Duan , C. Holtze , D. A. Weitz , A. D. Griffiths , C. A. Merten , Chem. Biol. 2008, 15, 427. 18482695 10.1016/j.chembiol.2008.04.004

[42] J. Zhai , Y. Liu , W. X. Huang , P. Wang , Y. Li , H. Li , A. H.‐H. Wong , X. Zhou , P. Chen , L. Wang , N. Yang , C. Chen , H. Chen , P.‐I. Mak , C.‐X. Deng , R. Martins , M. Yang , T.‐Y. Ho , S. Yi , H. Yao , Y. Jia , Nat. Commun. 2024, 15, 4363. 38778087 10.1038/s41467-024-48616-3 PMC11111680

[43] E. Brouzes , M. Medkova , N.. Savenelli , D. Marran , M. Twardowski , J. B. Hutchison , J. M. Rothberg , D. R. Link , N. Perrimon , M. L. Samuels , Proc. Nat. Acad. Sci. 2009, 106, 14195. 19617544 10.1073/pnas.0903542106 PMC2732882

[44] S. Avnet , G. D. Pompo , G. Borciani , T. Fischetti , G. Graziani , N. Baldini , Biomed. Mater. 2024, 19, 025033. 10.1088/1748-605X/ad2556 38306683

[45] H. Y. Jo , Y. Kim , H. W. Park , H. E. Moon , S. Bae , J. Kim , D. G. Kim , S. H. Paek , Exp. Neurobiol. 2015, 24, 235. 26412973 10.5607/en.2015.24.3.235 PMC4580751

[46] M. Ghasemi , T. Turnbull , S. Sebastian , I. Kempson , Int. J. Mol. Sci. 2021, 22, 12827. 34884632 10.3390/ijms222312827 PMC8657538

[47] F. Mittler , P. Obeïd , A. V. Rulina , V. Haguet , X. Gidrol , M. Y. Balakirev , Front. Oncol. 2017, 7, 293. 29322028 10.3389/fonc.2017.00293 PMC5732143

[48] J. R. Aguilar Cosme , D. C. Gagui , H. E. Bryant , F. Claeyssens , Front. Mol. Biosci. 2021, 8, 784962. 34869604 10.3389/fmolb.2021.784962 PMC8637197

[49] M. Zanoni , F. Piccinini , C. Arienti , A. Zamagni , S. Santi , R. Polico , A. Bevilacqua , A. Tesei , Sci. Rep. 2016, 6, 19103. 26752500 10.1038/srep19103 PMC4707510

[50] K. Vaidyanathan , C. Wang , A. Krajnik , Y. Yu , M. Choi , B. Lin , J. Jang , S.‐J. Heo , J. Kolega , K. Lee , Y. Bae , Sci. Rep. 2021, 11, 23285. 34857846 10.1038/s41598-021-02683-4 PMC8640073

[51] W. Abbaoui , S. Retal , B. El Bhiri , N. Kharmoum , S. Ziti , Inf. Med. Unlocked 2024, 46, 101475.

[52] C. Carini , A. A. Seyhan , J. Transl. Med. 2024, 22, 411. 38702711 10.1186/s12967-024-05067-0 PMC11069149

[53] L. Marques , B. Costa , M. Pereira , A. Silva , J. Santos , L. Saldanha , I. Silva , P. Magalhães , S. Schmidt , N. Vale , B. Costa , M. Pereira , A. Silva , J. Santos , L. Saldanha , I. Silva , P. Magalhães , S. Schmidt , N. Vale , Pharmaceutics 2024, 16, 332. 38543226 10.3390/pharmaceutics16030332 PMC10975777

[54] S. Park , S. E. Yoon , Y. Song , C. Tian , C. Baek , H. Cho , W. S. Kim , S. J. Kim , S. Cho , BMEMat 2024, e12128.

[55] I. Abd El‐Sadek , R. Morishita , T. Mori , S. Makita , P. Mukherjee , S. Matsusaka , Y. Yasuno , Sci. Rep. 2024, 14, 3366. 38336794 10.1038/s41598-024-53171-4 PMC10858208

[56] F. Yan , B. Mutembei , T. Valerio , G. Gunay , J.‐H. Ha , Q. Zhang , C. Wang , E. R. S. Mercyshalinie , Z. A. Alhajeri , F. Zhang , L. E. Dockery , X. Li , R. Liu , D. N. Dhanasekaran , H. Acar , W. R. Chen , Q. Tang , Biomed. Opt. Express, BOE 2024, 15, 2014. 38633082 10.1364/BOE.514079 PMC11019711

[57] O. Yasuhiko , K. Takeuchi , H. Yamada , Y. Ueda , Biomed. Opt. Express, BOE 2022, 13, 962. 35284178 10.1364/BOE.446622 PMC8884216

[58] L. Benning , A. Peintner , G. Finkenzeller , L. Peintner , Sci. Rep. 2020, 10, 11071. 32632214 10.1038/s41598-020-67960-0 PMC7338379

[59] K. Tröndle , G. Miotto , L. Rizzo , R. Pichler , F. Koch , P. Koltay , R. Zengerle , S. S. Lienkamp , S. Kartmann , S. Zimmermann , Int. J. Bioprint. 2022, 8, 528. 35702333 10.18063/ijb.v8i2.528 PMC9186384

[60] C.‐C. Chiang , R. Anne , P. Chawla , R. M. Shaw , S. He , E. C. Rock , M. Zhou , J. Cheng , Y.‐N. Gong , Y.‐C. Chen , Lab Chip 2024, 24, 3169. 38804084 10.1039/d4lc00197d PMC11165951

[61] C. Parent , K. Raj Melayil , Y. Zhou , V. Aubert , D. Surdez , O. Delattre , C. Wilhelm , J.‐L. Viovy , Lab Chip 2023, 23, 5139. 37942508 10.1039/d3lc00417a

[62] R. M. Haralick , K. Shanmugam , I. Dinstein , IEEE Trans. Syst. Man Cybern. 1973, SMC‐3, 610.

[63] F. Simon , M. C. Comes , T. Tocci , L. Dupuis , V. Cabeli , N. Lagrange , A. Mencattini , M. C. Parrini , E. Martinelli , H. Isambert , eLife 2025, 13, RP95485. 39819525 10.7554/eLife.95485 PMC11741518

[64] M. C. Ribeiro‐Dantas , H. Li , V. Cabeli , L. Dupuis , F. Simon , L. Hettal , A.‐S. Hamy , H. Isambert , iScience 2024, 27, 109736. 38711452 10.1016/j.isci.2024.109736 PMC11070693

[65] N. Sella , F. Guinot , N. Lagrange , L.‐P. Albou , J. Desponds , H. Isambert , npj Digit. Med. 2025, 8, 1. 39843957 10.1038/s41746-025-01431-6 PMC11754479

[66] M. Hall‐Beyer , GLCM Texture: A Tutorial v. 3.0, Mar. **2017**, https://ucalgary.scholaris.ca/items/8833a1fc‐5efb‐4b9b‐93a6‐ac4ff268091c.

[67] O. Rainio , J. Teuho , R. Klén , Sci. Rep. 2024, 14, 6086. 38480847 10.1038/s41598-024-56706-x PMC10937649

[68] L. Rösner , F. Walter , C. Ude , G. John , S. Beutel , Bioengineering 2022, 9, 762. 36550968 10.3390/bioengineering9120762 PMC9774925

[69] J. N. Rosenberg , N. C. Cady , Curr. Opin. Biotechnol. 2021, 71, 123. 34358978 10.1016/j.copbio.2021.07.004

[70] K. R. Kim , W. Yeo , BMEMat 2023, 1, e12047.

[71] H. Wu , Y. Yang , P. O. Bagnaninchi , J. Jia , Analyst 2018, 143, 4189. 30070264 10.1039/c8an00729b

[72] K. H. Tan , J. L. Y. Ang , A. S. K. Yong , S. Z. E. Lim , J. S. J. Kng , K. Liang , Biomed. Opt. Express 2024, 15, 6370. 39553864 10.1364/BOE.533339 PMC11563335

[73] G. Babakhanova , A. Agrawal , D. Arora , A. Horenberg , J. B. Budhathoki , J. P. Dunkers , J. Chalfoun , P. Bajcsy , C. G. Simon , J. Biomed. Mater. Res. 2023, 111, 1279. 10.1002/jbm.a.37528 36916776

[74] Y.‐T. Lai , Y.‐C. Li , Y.‐F. Chen , J.‐Y. Cheng , Microchem. J. 2025, 212, 113159.

[75] C. Buchou , K. Laud‐Duval , W. Van Der Ent , S. Grossetête , S. Zaidi , G. Gentric , M. Corbé , K. Müller , E. Del Nery , D. Surdez , O. Delattre , Cancers 2022, 14, 2327. 35565457 10.3390/cancers14092327 PMC9100622

[76] D. Ferraro , M. Serra , D. Filippi , L. Zago , E. Guglielmin , M. Pierno , S. Descroix , J.‐L. Viovy , G. Mistura , Lab Chip 2019, 19, 136. 10.1039/c8lc01182f 30484796

[77] D. Foreman‐Mackey , D. W. Hogg , D. Lang , J. Goodman , PASP 2013, 125, 306.

